# New Books

**Published:** 2009-12

**A Primer of Ecology with R**

M. Henry H. Stevens

New York:Springer, 2009. 388 pp. ISBN: 978-0-387-89881-0, $64.95

**Acute Exposure Guideline Levels for Selected Airborne Chemicals: Vol. 8**

Committee on Acute Exposure Guideline Levels; Committee on Toxicology; National Research Council

Washington, DC:National Academies Press, 2009. 434 pp. ISBN: 978-0-309-14515-2, $84.50

**Carbon Sinks And Climate Change Forests in the Fight Against Global Warming**

Colin A.G. Hunt

Northhampon, MA:Edward Elgar Publishing, 2009. 256 pp. ISBN: 978-1-84720-977-1, $110

**Contemporary Issues In International Environmental Law**

Malgosia Fitzmaurice

Northhampon, MA:Edward Elgar Publishing, 2009. 256 pp. ISBN: 978-1-84542-283-7, $99

**Ecological Economics, 2nd ed.: Principles and Applications**

Herman Daly, Joshua Farley

Washington, DC:Island Press, 2009. 488 pp. ISBN: 978-1-59726-681-9, $75

**Environmental Law Handbook, 20th ed.**

Daniel M. Steinway, et al.

Cincinnati, OH:ACGIH, 2009. 975 pp. ISBN: 978-1-60590-278-4, $99

**Global Sources of Local Pollution: An Assessment of Long-Range Transport of Key Air Pollutants to and from the United States**

Committee on the Significance of International Transport of Air Pollutants; National Research Council

Washington, DC:National Academies Press, 2009. 250 pp. ISBN: 978-0-309-14398-1, $42

**Governing The Environment: Salient Institutional Issues**

Albert Breton, Giorgio Brosio, Silvana Dalmazzone, Giovanna Garrone, eds.

Northhampon, MA:Edward Elgar Publishing, 2009. 978 pp. ISBN: 978-1-84720-397-7, $140

**Inorganic Chromium (III) Compounds**

World Health Organization

Geneva:WHO Press, 2009. 102 pp. ISBN: 978-9-2415-3076-7, $20

**International Handbook On The Economics of Energy**

Joanne Evans, Lester C. Hunt, eds.

Northhampon, MA:Edward Elgar Publishing, 2009. 848 pp. ISBN: 978-1-84720-352, $330

**Night Noise Guidelines for Europe**

WHO Regional Office for Europe

Geneva:WHO Press, 2009. 180 pp. ISBN: 978-9-2890-4173-7, $40

**Our Choice: A Plan to Solve the Climate Crisis**

Al Gore

London:Bloomsbury Publishing, 2009. 416 pp. ISBN: 978-0-7475-9098-9, $20.90

**Technonatures: Environments, Technologies, Spaces, and Places in the 21st Century**

Damian F. White, Chris Wilbert, eds.

Waterloo, ON:Wilfrid Laurier University Press, 2009. 282 pp. ISBN: 978-1-55458-150-4, $38.95

